# MicroRNAs underlie plasmacytoid dentritic cell dysfunction in systemic sclerosis

**DOI:** 10.1186/1479-5876-10-S3-P54

**Published:** 2012-11-28

**Authors:** Jasper C Broen, Lenny van Bon, Nick P Rossen, Marzia Rossato, Timothy RDJ Radstake

**Affiliations:** 1 Dept. of Rheumatology, Clinical Immunology and Translational Immunology, University Utrecht Medical Center, Utrecht, the Netherlands

## Background

Systemic sclerosis (SSc) is an autoimmune disease characterized by fibrosis of the skin and internal organs that carries a high burden of morbidity and mortality. Immune system dysfunction has been undisputedly demonstrated to underlie SSc pathogenesis. In particular, pDCs not only circulate in very high number in SSc patients but also secrete high concentrations of specific chemokines that directly drive various features of SSc, including endothelial dysfunction and fibroblast activation.

## Materials and methods

Plasmacytoid dendritic cells (pDCs) were isolated from whole blood of early diffuse SSc, late diffuse SSc, limited SSc patients or healthy controls. The genome-wide profiling of microRNAs (miRNA) was performed on pDC total RNA by using the Illumina miRNA Profiling Array. Genes targeted by differentially expressed miRNAs were identified thanks to a combination of Miranda, Mirtar and MirPath prediction algorithms.

## Results

On the quest to decipher why pDCs appear so abundantly in SSc and their activity is deregulated, miRNA profiling was performed. miRNA screening demonstrated that seven miRNAs were significantly over- and two were under-expressed in pDCs from patients with early diffuse SSc compared to late diffuse disease, limited SSc or healthy controls and this profile perfectly correlated with pDC abundance in the different disease stages. According to Miranda, Mirtar and MirPath prediction algorithms, this set of 9 miRNAs is predicted to influence a vast number of target genes, mostly involved in three molecular pathways: 1) leukocyte apoptosis, 2) epidermal growth factor EGFR (ErbB) signaling and 3) WNT signaling. Interestingly, the emergence of the leukocyte apoptosis pathway might provide a preliminary hint on why pDCs are expanded early in SSc. On the other side, ErbB and WNT signaling are ultimately involved in immune response regulation and often linked to the malfunctioning of the immune system in SSc.

## Conclusions

Altogether these unique observations suggest that SSc-associated miRNAs could strongly impact on cellular pathways involved in SSc pathology, and therefore they would provide an important target for disease prediction and therapeutic approaches.

